# Sex-Specific B Cell and Anti-Myelin Autoantibody Response After Peripheral Nerve Injury

**DOI:** 10.3389/fncel.2022.835800

**Published:** 2022-04-14

**Authors:** Hee Jong Lee, Albert G. Remacle, Swathi K. Hullugundi, Jennifer Dolkas, Jake B. Leung, Andrei V. Chernov, Tony L. Yaksh, Alex Y. Strongin, Veronica I. Shubayev

**Affiliations:** ^1^Department of Anesthesiology, University of California, San Diego, La Jolla, CA, United States; ^2^VA San Diego Healthcare System, La Jolla, CA, United States; ^3^Department of Anesthesiology & Pain Medicine, Hanyang University, Seoul, South Korea; ^4^Infectious and Inflammatory Disease Center, Sanford Burnham Prebys Medical Discovery Institute, La Jolla, CA, United States

**Keywords:** autoantibody, neuropathic pain, sex, sexual dimorphism, myelin, autoantigen

## Abstract

Immunotherapy holds promise as a non-addictive treatment of refractory chronic pain states. Increasingly, sex is recognized to impact immune regulation of pain states, including mechanical allodynia (pain from non-painful stimulation) that follows peripheral nerve trauma. This study aims to assess the role of B cells in sex-specific responses to peripheral nerve trauma. Using a rat model of sciatic nerve chronic constriction injury (CCI), we analyzed sex differences in (i) the release of the immunodominant neural epitopes of myelin basic protein (MBP); (ii) the levels of serum immunoglobulin M (IgM)/immunoglobulin G (IgG) autoantibodies against the MBP epitopes; (iii) endoneurial B cell/CD20 levels; and (iv) mechanical sensitivity behavior after B cell/CD20 targeting with intravenous (IV) Rituximab (RTX) and control, IV immunoglobulin (IVIG), therapy. The persistent MBP epitope release in CCI nerves of both sexes was accompanied by the serum anti-MBP IgM autoantibody in female CCI rats alone. IV RTX therapy during CD20-reactive cell infiltration of nerves of both sexes reduced mechanical allodynia in females but not in males. IVIG and vehicle treatments had no effect in either sex. These findings provide strong evidence for sexual dimorphism in B-cell function after peripheral nervous system (PNS) trauma and autoimmune pathogenesis of neuropathic pain, potentially amenable to immunotherapeutic intervention, particularly in females. A myelin-targeted serum autoantibody may serve as a biomarker of such painful states. This insight into the biological basis of sex-specific response to neuraxial injury will help personalize regenerative and analgesic therapies.

## Introduction

Neuropathic pain is a persistent, treatment-refractory condition that may arise after peripheral nervous system (PNS) trauma. Non-addictive analgesic strategies to manage neuropathic pain have often centered on neuroimmune modulation (Myers et al., 2006; Scholz and Woolf, 2007; Myers and Shubayev, 2011), including selective targeting of the adaptive immune system (Moalem et al., 2004; Kleinschnitz et al., 2006; Costigan et al., 2009; Kim and Moalem-Taylor, 2011b; Austin et al., 2012; Liu et al., 2012, 2015; Draleau et al., 2014). Comparative analysis of male and female mice with PNS trauma revealed a sexually dimorphic (female-prevalent) role of T-cell activity in regulating the maintenance of mechanical allodynia (Sorge et al., 2015). However, the importance of the overactive adaptive immune system, particularly the activity of T helper (Th)1 and Th17 cells, in mechanical allodynia and other phenotypes of neuropathic pain, has previously been established mainly in male rodents (Moalem et al., 2004; Kleinschnitz et al., 2006; Costigan et al., 2009; Kim and Moalem-Taylor, 2011b; Austin et al., 2012; Liu et al., 2012, 2015; Draleau et al., 2014). A deeper mechanistic understanding of sexual dimorphism in adaptive immune regulation of neuropathic pain is necessary to reconcile these paradoxical findings. The identity and activity of cognate antigens involved in “sterile” inflammation of PNS trauma, and the resulting B- and T-cell recruitment and function could be distinct in male and female PNS.

Myelin basic protein (MBP) is an intrinsically unstructured cationic protein of the myelin sheath and a putative neural autoantigen (Kadlubowski and Hughes, 1979; Boggs, 2006). Immunodominant MBP epitopes are concealed within intact myelin and, when released by proteases, contribute to pathogenesis of autoimmune demyelinating conditions, multiple sclerosis, and Guillain-Barre syndrome (Kadlubowski and Hughes, 1979; Boggs, 2006). Because PNS myelin insulates primary A-type mechanosensory afferents, we have implicated the loss of myelin integrity after a focal PNS trauma and the resulting MBP epitope release in the pathogenesis of mechanical allodynia due to the localized autoimmune remodeling of A-afferents (Kobayashi et al., 2008; Liu et al., 2012; Hong et al., 2017; Chernov et al., 2018).

Specifically, in a female rat model of sciatic nerve trauma, including chronic constriction injury (CCI), we have observed the release of the central immunodominant MBP epitopes, including 84–104 and 69–86 (Kobayashi et al., 2008; Liu et al., 2012; Hong et al., 2017; Chernov et al., 2018). An intra-sciatic adjuvant-free bolus injection of the synthetic peptides encoding rat or human MBP(84–104) and/or MBP(69–86) peptides is sufficient to induce robust, persistent, and T-cell-dependent mechanical allodynia (Liu et al., 2012; Ko et al., 2016; Hong et al., 2017; Chernov et al., 2018). Blockade of the MBP epitope release by matrix metalloproteinase (MMP) inhibition (Kobayashi et al., 2008; Liu et al., 2012; Hong et al., 2017; Chernov et al., 2018) and neutralization of the epitope activity by active immunization with the altered MBP ligand (Perera et al., 2015) attenuate CCI-induced mechanical allodynia.

Remarkably, the pro-allodynic MBP(84–104) activity emerged as sexually dimorphic (female-specific) in mice. Equal-dose intra-sciatic MBP(84–104) injection in mice of both sexes resulted in female-specific mechanical allodynia (Chernov et al., 2020). The nerve injection site, ipsilateral dorsal root ganglia (DRG), and spinal cord display a major sex-specific transcriptional reprogramming induced by MBP(84–104) relative to a scrambled (SCR) peptide or buffer vehicle (Chernov et al., 2020). Among the numerous specific changes was female-prevalent T- and B-cell receptor signaling activity (Chernov et al., 2020).

Many chronic pain states (Fillingim et al., 2009; Nahin, 2015) and autoimmune diseases (Whitacre et al., 1999; Voskuhl, 2011) are female-prevalent public health issues. While the relationship between sex, pain, and autoimmunity is not well understood, persistent pain associated with autoimmune diseases, such as rheumatoid arthritis, has been attributed to the autoantibody-mediated sensitization of primary afferent neurons (Sorkin, 2000; Bennett and Vincent, 2012; McMahon et al., 2015; Goebel, 2016; Wigerblad et al., 2016; Goebel et al., 2021). In addition to the known autoimmune conditions, cases of complex regional pain syndrome (CRPS) that develop after limb trauma have been considered to have autoimmune pathogenesis (Goebel et al., 2010, 2011, 2017; Kohr et al., 2011; Goebel and Blaes, 2013; Li et al., 2014, 2018; Tekus et al., 2014; Littlejohn, 2015; Goebel, 2016; Guo et al., 2017), and patients with CRPS have shown to benefit from intravenous (IV) Rituximab (RTX) immunotherapy (Goebel and Blaes, 2013).

The RTX is a chimeric murine-human monoclonal antibody against the cell surface CD20 glycoprotein expressed by immature and mature B cells (Weiner, 2010). Developed to treat B-cell lymphoma through the complement- and antibody-dependent B-cell cytotoxicity, IV RTX has increasingly been used to manage rheumatoid arthritis, systemic lupus erythematosus, and other autoimmune states (Kazkaz and Isenberg, 2004). Given that IV RTX demonstrated analgesic potency in patients with CRPS (Goebel and Blaes, 2013) and anti-myelin-associated glycoprotein (MAG) immunoglobulin M (IgM) demyelinating neuropathy (Dalakas et al., 2009) and has shown efficacy in rat disease models (Novotny et al., 2012; Li et al., 2013; Takahashi et al., 2017), in this study, we used a rat sciatic nerve CCI mononeuropathy model to assess sex differences in MBP epitope release, anti-MBP autoantibody generation, and anti-allodynic potency of IV RTX therapy for a focal PNS trauma.

## Materials and Methods

### Reagents and Antibodies

General reagents were purchased from Sigma-Aldrich and Thermo Fisher Scientific. Human MBP (18.5 kDa isoform) was purchased from Meridian Life Science. The MBP(84–104) (ENPVVHFFKNIVTPRTPPPSQ) based on human MBP sequence (AAH08749, GenBank) and SCR (EFPHIKVTVVTPRNGFPNSPP) peptides, i.e., *N*-terminally biotinylated and *C*-terminally amidated peptides, were synthesized by GenScript. Rabbit polyclonal anti-CD20 antibody was obtained from Abcam (ab85809), mouse monoclonal anti-β-actin antibody was obtained from Sigma (A53166), and rabbit anti-degraded (d)MBP antibody, generated against the synthetic peptide encoding guinea pig MBP(69–86), was obtained from EDM-Millipore (AB5864). FluoroMyelin 488 (F34651) was obtained from Molecular Probes. For ELISA, HRP-conjugated goat anti-rat IgM (3020-05) and anti-rat immunoglobulin G (IgG) (112-035-175, Jackson ImmunoResearch), a 3,3′,5,5′-tetramethylbenzidine substrate (TMB/E; Surmodics), and BSA (an IgG and protease-free, 30% solution, United States Biological) were used.

### Chronic Constriction Injury

Sprague-Dawley rats (Envigo, 8-week-old, female and male) were housed in temperature-controlled cages with a 12-h light-dark cycle and free access to food and water (H_2_O). The procedures and behavioral testing were performed during the light cycle. Animals were randomly assigned to the experimental groups based on sex. Under 3% isoflurane anesthesia, the common sciatic nerve was unilaterally exposed at the mid-thigh level with sterile technique. CCI was produced by tying three loosely constrictive chromic gut ligations around the sciatic nerve (Bennett and Xie, 1988). The Sham operation included nerve exposure with no injury. Animals were sacrificed by intraperitoneal Euthasol (100–150 mg/ml; Virbac Animal Health) after isoflurane inhalation anesthesia. Spleen and sciatic nerve tissues were harvested, snap-frozen in liquid nitrogen (N_2_), and stored at −80°C for immunoblotting or transcardial perfusion using 4% paraformaldehyde (PFA) in 0.2 M phosphate buffer for immunostaining. For ELISA, 1–2 ml blood aliquots were collected before CCI (day 0) and after days 2, 14, and 28 post-CCI by cardiac puncture in tubes without anti-coagulant, allowed to clot for 30 min at room temperature, and centrifuged (2,000 × *g*; 10 min; 4°C), and the supernatant (serum) was collected and stored at −80°C. Animal procedures were performed according to the protocols approved by the Institutional Animal Care and Use Committee at the Veterans Affairs San Diego Healthcare System, the Public Health Service Policy on Humane Care and Use of Laboratory Animals, and the ethical guidelines of the International Association for the Study of Pain.

### Drugs and Drug Delivery

Rituxan^®^ (rituximab, RTX) was obtained from Genentech. IV immunoglobulin (IVIG) Gamunex-C (immune globulin injection 10%) was purchased from Grifols. RTX and IVIG were diluted in phosphate buffer solution (PBS; Steris Labs). At day 7 post-CCI, RTX (10 mg/kg, *n* = 18 [female: *n* = 9 and male: *n* = 9]), IVIG (10 mg/kg, *n* = 12 [male: *n* = 6 and female: *n* = 6]), or PBS vehicle (*n* = 12 [male: *n* = 6 and female: *n* = 6]) were administered IV through the tail vein in the same volume (10 μl) using a 27-gauge needle.

### Von Frey Testing

Withdrawal threshold to non-noxious mechanical stimuli was assessed by von Frey testing using Dixon’s up-down method (Chaplan et al., 1994). Rats were acclimated to the Plexiglas compartments with 6-mm wire grid bottom and habituated to the environment for 2 days prior to baseline testing. Baseline values were established for 3 consecutive days before surgery and then up to daily between 2 and 16 days post-CCI, as indicated previously. Mechanical stimuli were applied using von Frey filaments (0.4–15.2 g, Stoelting, Wood Dale, IL, United States) perpendicularly on the plantar area of the hind paw innervated by the sciatic nerve. The stimuli were applied for 2 s, with a 10-s interval between each stimulus or until the rat was stable from the pain. The responses were recorded as positive if the paw was rapidly withdrawn. The 50% withdrawal threshold was calculated according to Dixon’s up-down method. Testing was performed at fixed times between 8:00 a.m. and 2:00 p.m. by experimenters blinded to the treatment conditions. Data were unblinded during data analysis.

### Immunohistochemistry

After transcardial perfusion with 4% PFA, tissues (e.g., sciatic nerves and spleen) were excised, postfixed in 4% PFA for 16–18 h, and rinsed in 0.2 M PBS (pH 7.4). Tissues for OCT went through 15–30% sucrose gradient before embedding in the optimal cutting temperature compound (Sakura Finetek) or processed for paraffin and cut into transverse, 10-μm-thick sections (Kobayashi et al., 2008; Liu et al., 2012; Ko et al., 2016; Hong et al., 2017). Deparaffination was completed in xylene, followed by rehydration in graded ethanol and PBS. Non-specific binding was blocked with 5% goat serum for 30 min at room temperature, followed by polyclonal rabbit anti-CD20 (ab85809, Abcam, 1:200) or dMBP (AB5864, EDM-Millipore, 1:2,000) antibody application at 4°C overnight. After rinsing in PBS, the sections were incubated with the goat anti-rabbit Alexa-conjugated secondary antibody (red, Thermo Fisher Scientific) for 1 h at room temperature. In selected frozen sections, FluoroMyelin 488 (F34651, Molecular Probes, 1:500) was applied for 20 min at room temperature. Slides were mounted in SlowFade Gold anti-fade reagent containing 4′,6-diamidino-2-phenylindole (DAPI; blue, Thermo Fisher Scientific). Staining specificity was confirmed by a primary antibody omission. The images were acquired by using an all-in-one fluorescence microscope BZ-X700 (Keyence, Itasca, IL, United States) and Leica fluorescence microscope.

### Immunoblotting

Tissue (e.g., sciatic nerves and spleen) extracts were prepared in TBS and supplemented with 1% Triton X-100, 10% glycerol, 0.1% SDS, 5 mM EDTA, and Halt Protease and Phosphatase Inhibitor Cocktail (Thermo Fisher Scientific #11861281). Insoluble material was removed by centrifugation (14,000 × *g*; 15 min). Extract aliquots (50–150 μg of total protein) were separated by 15% Tris-glycine SDS-gel electrophoresis (Bio-Rad). Separated proteins were transferred onto a PVDF membrane. The membrane was blocked with 5% non-fat milk (Bio-Rad) and incubated for 16–18 h at 4°C with the rabbit polyclonal anti-CD20 antibody (ab85809, Abcam), followed by incubation for 1 h at room temperature with the rabbit-specific HRP-conjugated goat secondary antibody (Cell Signaling; 1:2,000 dilution). Three washes in TBS/T at room temperature were performed after each step. The membranes were re-probed using a β-actin antibody as a loading control (A53166, Sigma). The blots were developed using the SuperSignal West Dura Extended Duration Substrate kit (Thermo Fisher Scientific) and then digitized and quantitated using Image J.

### Enzyme-Linked Immunosorbent Assay

Serum was tested by the anti-MBP(84–104) epitope IgG and IgM ELISA (Remacle et al., 2018a). The wells of a 96-well Maxisorp ELISA plate were coated with ExtrAvidin (3 μg/ml in 0.125 ml, 15 mM bicarbonate buffer, pH 9.6) or BSA (3 μg/ml, control) for 18 h at 4°C. The wells were blocked for 1 h at 37°C using 1% IgG and protease-free BSA (0.4 ml) in 50 mM Tris–HCl buffer, pH 7.8, containing 1 M NaCl and 0.1% Tween-20 (TBS/T). Incubation with the biotin-labeled MBP(84–104) and SCR peptides (5 μg/ml in 0.1 ml TBS/T-1% BSA, each) continued at 4°C for 16–18 h. Serum samples (1:50 dilution in 0.1 ml TBS/T-1% BSA) were allowed to bind to the wells for 3 h. The secondary HRP-conjugated goat anti-rat IgG or IgM antibodies (1:10,000 dilution in 0.1 ml TBS/T-1% BSA, each) were added for 1 h. Six washes (5 min each; 500–700 rpm) in TBS/T at room temperature were performed after each step. After a wash with H_2_O, the TMB/E (0.1 ml) was added to the wells. The reaction was stopped using 1 M sulfuric acid (H_2_SO_4_; 0.1 ml) and estimated at *A*_450_ using a plate reader. The *A*_450_ values for MBP(84–104) peptide were calculated relative to the SCR peptide. The threshold values for the MBP (*A*_450_ = 0.298) and SCR (*A*_450_ = 0.237) peptides were determined, as described previously (Remacle et al., 2018a). Data represent means ± SE from 3 individual experiments performed in triplicate.

### Statistical Analyses

GraphPad Prism version 6.0 software (Synergy Software) was used to conduct two-way repeated measures ANOVA or a one-way ANOVA with the Bonferroni or Holm-Sidak *post-hoc* test or Mann-Whitney *U*-test or Wilcoxon rank-sum test and Kruskal–Wallis non-parametric ANOVA implementing Dunn’s procedure, as detailed in the figure legends. Data are presented as the mean ± SEM, and differences are considered statistically significant at *p* < 0.05.

## Results

**FIGURE 1:**
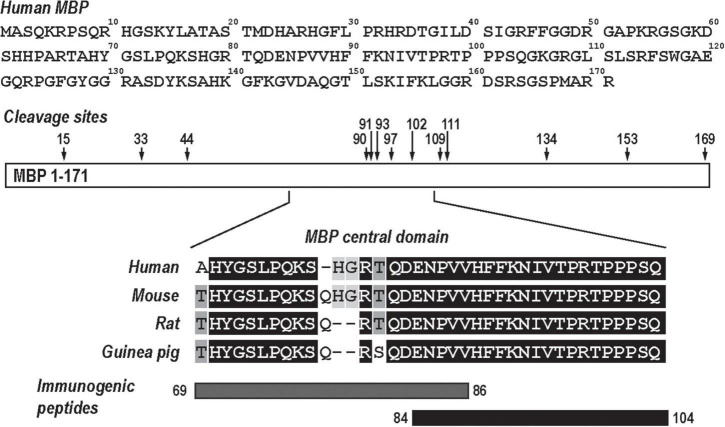
Myelin basic protein (MBP), a neural autoantigen (a schematic diagram). The human MBP sequence (GenBank, AAH08749) has multiple proteolytic cleavage sites (arrows). Sequence alignment of the evolutionary conserved central region of MBP and its immunogenic peptides, MBP(69–86) and MBP(84–104).

The MBP is an intrinsically unstructured myelin protein with multiple proteolytic cleavage sites (Figure 1). A conserved central domain of MBP conceals dominant autogenic epitopes released by proteolysis as peptides, including MBP(69–86) and MBP(84–104). Synthetic MBP(69–86) and MBP(84–104) peptides, but not *N*-terminal MBP or SCR peptides, produce mechanical allodynia (Kobayashi et al., 2008; Liu et al., 2012; Perera et al., 2015; Ko et al., 2016; Hong et al., 2017; Chernov et al., 2018) in a sex-specific manner (Chernov et al., 2020) and present as valuable tools in assessing sex differences in autoantigenic epitope and autoantibody production after PNS trauma (Figure 1).

### Myelin Basic Protein Epitope Release in Chronic Constriction Injury Nerve of Both Sexes

**FIGURE 2:**
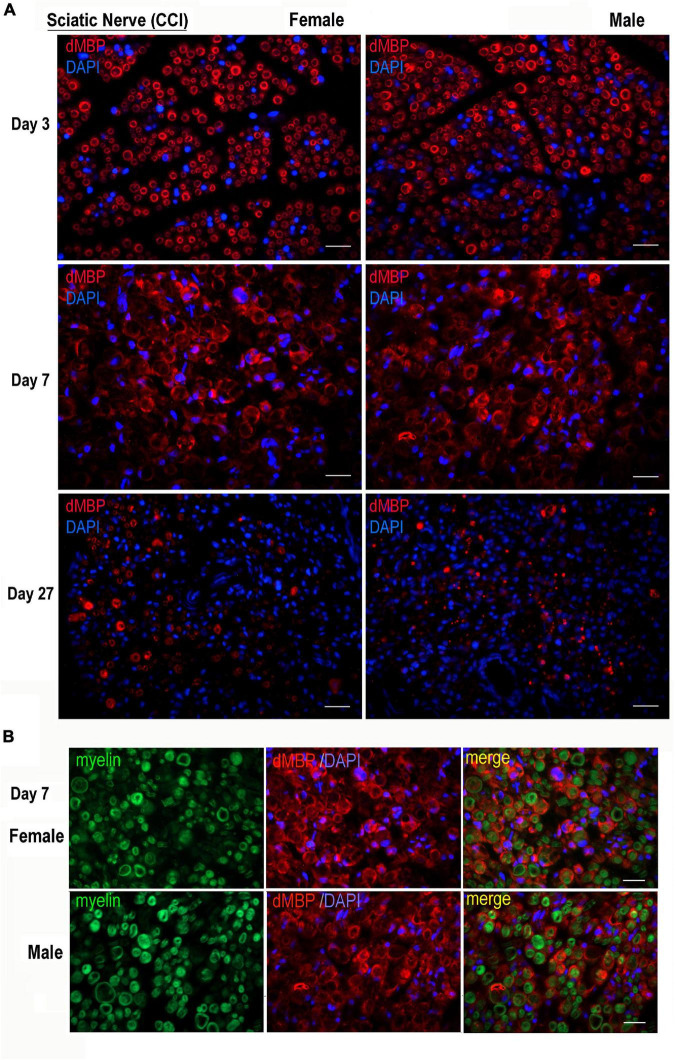
Degraded MBP epitope release in female and male chronic constriction injury (CCI) nerve. **(A)** Degraded dMBP immunoreactivity (AB5864, EDM-Millipore, red) in female and male sciatic CCI nerves (days 3, 7, and 27). 4′,6-diamidino-2-phenylindole (DAPI) nuclear stain, blue. Representative of *n* = 3/group. Scale bars, 50 μm. **(B)** FluoroMyelin (green) and dMBP immunostaining (red) in demyelinating female and male sciatic CCI nerve (day 7); note: dMBP in myelin ovoid and perimyelin structures. DAPI nuclear stain (blue). Representative of *n* = 3/group. Scale bars, 50 μm.

The AB5864 EDM-Millipore antibody generated against guinea pig MBP(69–86) peptide detects the degraded (d)MBP, but not intact MBP, as a marker of demyelinating lesions (Matsuo et al., 1997), including the one in the PNS (Kobayashi et al., 2008; Liu et al., 2012; Hong et al., 2017; Chernov et al., 2018). Female and male nerves were immunostained for dMBP at days 3, 7, and 27 post-CCI, corresponding to early and peak demyelination and remyelination, respectively (Figure 2). Total myelin was identified with FluoroMyelin. In nerves of both sexes, dMBP was observed in myelin sheaths (donut structures) and myelinating Schwann cells (crescent structures) at the analyzed time points (Figure 2A). In addition, dual-reactivity with FluoroMyelin highlighted dMBP in perimyelin and myelin ovoids in demyelinating nerves (Figure 2B, day 7). There was no significant difference observed in the pattern or the level of dMBP release between sex at the analyzed time points.

### Female-Specific Increase in Serum Anti-myelin Basic Protein Autoantibody Post-chronic Constriction Injury

**FIGURE 3:**
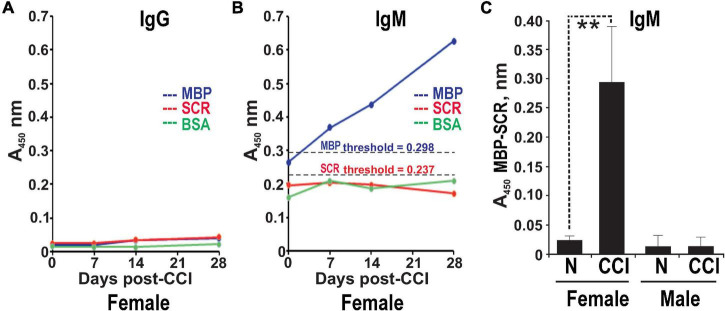
Female-specific serum anti-MBP autoantibodies post-CCI. The anti-MBP(84–104) immunoglobulin G (IgG) **(A)** and immunoglobulin M (IgM) **(B)** ELISA in the serum of female rats post-CCI. The biotin-labeled MBP(84–104) (MBP, blue) and scrambled (SCR, red) peptides or BSA (control, green) were immobilized on the ExtrAvidin-coated wells. Serum aliquots (*n* = 4–8/group) before CCI (days) and post-CCI (days 7, 14, and 28) were allowed to bind to the peptides. The bound antibodies were detected using HRP-conjugated anti-rat IgM or IgG and a 3,3′,5,5′-tetramethylbenzidine substrate (TMB/E). The threshold values for MBP (*A*_450_ = 0.298) and SCR (*A*_450_ = 0.237) (black dotted lines) were determined earlier (Remacle et al., 2018a). **(C)** Anti-MBP(84–104) IgM in the serum of female and male rats post-CCI. Serum aliquots before CCI (day 0) and post-CCI (day 28) were allowed to bind to the immobilized MBP(84–104) and SCR peptides. The antibodies were detected using HRP-conjugated anti-rat IgM and a TMB/E. The *A*_450_ values for the MBP peptide were calculated relative to the SCR peptide (*A*_450_ MBP-SCR). Means ± SE from *n* = 4/group and 3 individual experiments performed in triplicate. **p < 0.01.

The serum ELISA using immobilized human MBP(84–104) peptide effectively detects specific IgG and IgM autoantibodies in patients with multiple sclerosis (Remacle et al., 2018a). It was used to analyze male and female rats pre-CCI (day 0) and at days 7, 14, and 28 post-CCI in the same rat cohorts. IgG against MBP(84–104) was not detectable in the rat serum pre- and post-CCI of either sex (e.g., female shown in Figure 3A). A gradual and time-dependent increase in IgM against MBP(84–104) was observed starting at day 7 until at least day 28 post-CCI in females (Figure 3B). There was no IgM reactivity against the SCR peptide or BSA controls detected at any time point (Figure 3B). At day 28 post-CCI, the anti-MBP(84–104) IgM autoantibodies were elevated ∼7-fold relative to pre-CCI in females (day 0, Figure 3C). There was no IgM against MBP(84–104) detected in the male serum pre- or post-CCI (day 28, Figure 3C). All *A*_450_ values represent the difference between MBP(84–104) and SCR peptides. These data indicate that production and/or secretion of anti-MBP IgM begins at day 7 post-CCI and is female-specific.

### Sex-Specific Effect of Intravenous Rituximab Therapy in Chronic Constriction Injury-Induced Mechanical Allodynia

**FIGURE 4:**
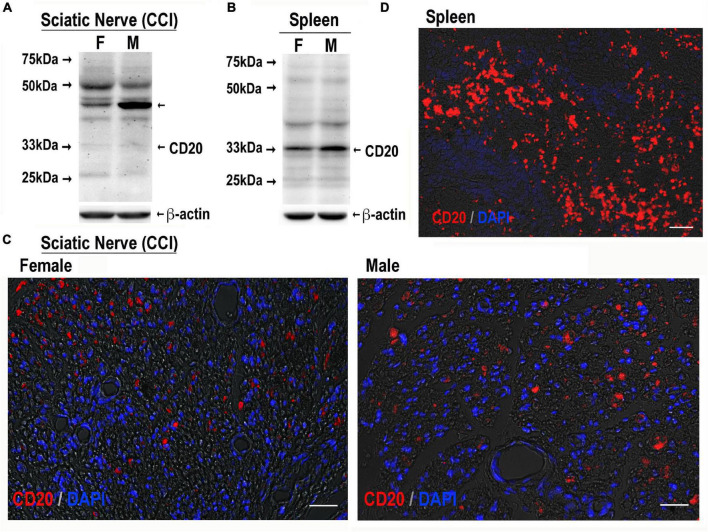
CD20-reactive cells in CCI nerve of both sexes. CD20 immunoblotting in female and male sciatic nerve [50 μg of protein, each, **(A)**] or spleen [80 μg of protein, each, **(B)**] post-CCI (day 17); β-actin, loading control. Representative of *n* = 3/group. CD20 immunostaining (red) in female and male sciatic nerve (day 17 post-CCI, **(C)**; spleen **(D)**, positive control. DAPI nuclear stain (blue). Representative of *n* = 3/group. Scale bars, 50 μm.

**FIGURE 5:**
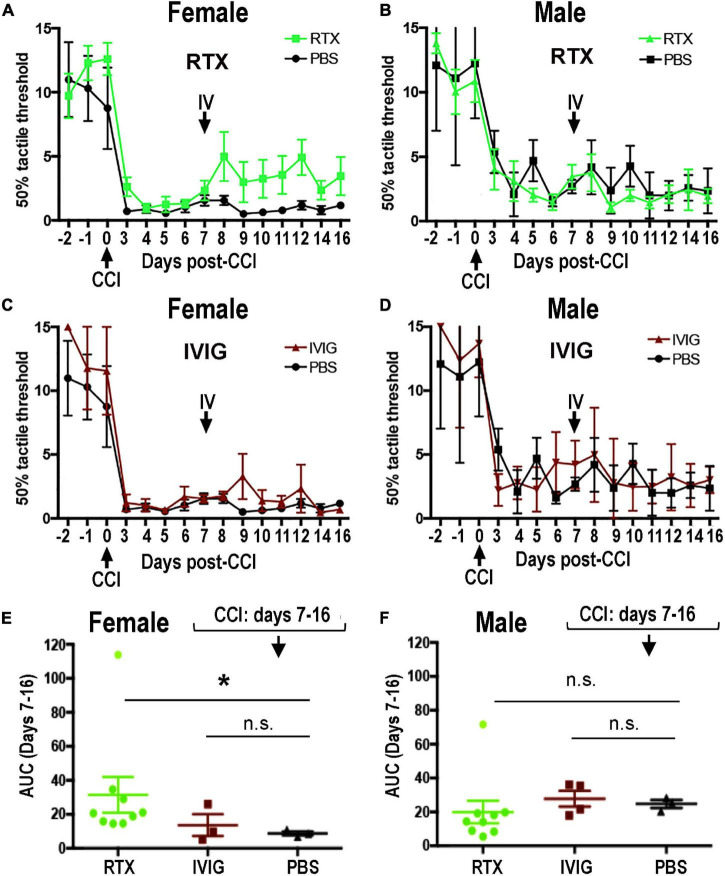
Female-specific effect of intravenous (IV) Rituximab (RTX) therapy on pain-like behavior post-CCI. Von Frey testing after bolus IV RTX [10 mg/kg in 10 μl phosphate buffer solution (PBS)], intravenous immunoglobulin (IVIG)/Gamunex-C, 10 mg/kg in 10 μl PBS) or PBS (10 μl) administered once at day 7 post-CCI. The mean mechanical withdrawal thresholds (gram force, g) ± SEM of CCI-RTX in females **(A)** and males **(B)**, CCI IVIG in females **(C)** and males **(D)**, and CCI-PBS in females **(A,C)** and males **(B,D)**. Two-way ANOVA and Holm-Sidak *post-hoc* test at 10 days after RTX vs. PBS in females (*p* = 0.066). Area-under-the-curve (AUC) analysis of female **(E)** and male **(F)** von Frey data from days 7–16 post-CCI corresponding to **(A,C)** (female) and **(B,D)** (male). Non-parametric Mann-Whitney *U*-test, comparing to day 7 baseline (*p = 0.0091). The data are based on *n* = 18 [female: *n* = 9 and male: *n* = 9] in RTX group, *n* = 12 [male: *n* = 6 and female: *n* = 6] in IVIG group, and *n* = 12 [male: *n* = 6 and female: *n* = 6] in PBS group.

Upon a cognate antigen encounter, such as MBP, B cells enter distinct phenotypic fates to produce IgG/IgM and prime Th cells. B cells are known to infiltrate both female and male sciatic nerves, including at days 3–14 post-CCI (Kim and Moalem-Taylor, 2011a; Liu et al., 2012; Chernov et al., 2020; Kalinski et al., 2020). Because CD20 is a B-cell-specific surface glycoprotein, effectively targeted by IV RTX therapy in autoimmune diseases (Kazkaz and Isenberg, 2004; Weiner, 2010), CRPS (Goebel and Blaes, 2013), and IgM demyelinating neuropathy (Dalakas et al., 2009), we confirmed the presence of CD20-reactive cells in CCI nerves of both sexes (Figure 4) and analyzed the anti-allodynic potency of IV RTX post-CCI (Figure 5).

#### B Cells Present in Male and Female Chronic Constriction Injury Nerves

CD20 immunoblotting (Figures 4A,B) and immunostaining (Figures 4C,D) were conducted in female and male nerves at days 7–21 post-CCI using the spleen as a positive control. The 33 kDa CD20 monomer band was readily identified in the spleen and faintly detectable in CCI nerves in both sexes (Figure 4B). Several, presumably non-specific, bands were observed in the spleen and nerve samples of both sexes, including the ∼46 kDa band selectively enriched in male nerves. CD20-positive cells showed no apparent difference between sex in the number or distribution in CCI nerves (Figure 4C). Their small and round morphology (Figure 4C) was the characteristic of B cells observed in clusters in the spleen (Figure 4D).

#### Female-Specific Effect of Intravenous Rituximab Therapy Post-chronic Constriction Injury

The RTX, a human-mouse chimeric mAb against a human CD20 antigen, has shown efficacious in rat disease models (Novotny et al., 2012; Li et al., 2013; Takahashi et al., 2017). To assess the anti-allodynic potency of IV RTX post-CCI, female and male rats received a single bolus IV RTX injection (10 mg/kg in 10 μl PBS, *n* = 18 [female: *n* = 9 and male: *n* = 9]) at day 7 post-CCI. IVIG, a preparation of heterogeneous polyclonal serum IgG against a broad range of antigens (10 mg/kg in 10 μl PBS, *n* = 12 [male: *n* = 6 and female: *n* = 6]), and PBS vehicle (10 μl, *n* = 12 [male: *n* = 6 and female: *n* = 6]) were used as controls. Withdrawal threshold to non-noxious mechanical stimuli was assessed by von Frey testing for 3 consecutive days before CCI and then up to daily post-CCI, as shown in Figure 5.

A stable decline in paw withdrawal thresholds to von Frey stimuli was observed at day 3 post-CCI in all experimental groups, corresponding to mechanical allodynia (Figures 5A–F). IV RTX therapy increased withdrawal thresholds relative to IV PBS in females (*p* = 0.066, two-way ANOVA, Figure 5A). In males, partly due to the variable withdrawal threshold values, there was no significant effect of IV RTX relative to PBS treatment (*p* > 0.05, Figure 5B). IVIG therapy produced no significant change in the withdrawal thresholds relative to IV PBS (*p* > 0.05) in female (Figure 5C) or male (Figure 5D) rats post-CCI. According to the analyses of the area under the curve (AUC) between day 7 and day 16 post-CCI, the anti-allodynic effect of IV RTX was significant relative to IV PBS in female rats (*p* = 0.0091, non-parametric Mann-Whitney *U*-test and Kruskal–Wallis test, Figure 5E) but not in male rats (Figure 5F). Contralateral to CCI, hind paws displayed no mechanical hypersensitivity in all experimental groups (data not shown). A single bolus IV RTX, IVIG, or PBS treatment produced no significant effect on the nerve or spleen CD20 levels (Supplementary Figure 1) or serum anti-MBP IgM levels (data not shown). We concluded that sex specificity of B-cell function after a focal PNS trauma can be therapeutically targeted to manage neuropathic pain, specifically in females.

## Discussion

Neuroinflammation persisting after acute recovery from PNS trauma has been repeatedly shown to mediate the development and maintenance of neuropathic pain through the coordinated activity of immune and immunocompetent glial cells at every level of the damaged sensory neuraxis (Myers et al., 2006; Scholz and Woolf, 2007; Myers and Shubayev, 2011). Increasingly, evidence is accumulating in support of sex-specific immune, genetic, and hormonal mechanisms of pain (Greenspan et al., 2007; Mogil, 2012; Sorge et al., 2015; Boerner et al., 2018; Gregus et al., 2021). The transition from innate to adaptive immune mediation of mechanical allodynia caused by PNS injury is thought to occur preferentially in females, as males maintain pain through activation of innate immune activity of spinal microglia and DRG macrophages (Mogil, 2012; Sorge et al., 2015; Yu et al., 2020). Since the role of Th cells in neuropathic pain has been repeatedly reported in male rodents (Moalem et al., 2004; Kleinschnitz et al., 2006; Costigan et al., 2009; Kim and Moalem-Taylor, 2011b; Austin et al., 2012; Liu et al., 2012, 2015; Draleau et al., 2014), more research is required to determine sexually monomorphic and dimorphic processes driving engagement of the adaptive immune arm in PNS injury and pain.

This study adds to the growing evidence of sex-specific immune response to PNS injury. Despite relatively comparable endoneurial levels of autogenic MBP epitopes and B cells in the damaged PNS of both sexes, the circulating anti-MBP autoantibody and anti-allodynic effect of B cell/CD20-targeting using RTX therapy were sexually dimorphic (both female-specific). It is, however, likely that these two findings pointing at sex-specific B-cell function in response to PNS injury are unrelated, as discussed below.

Despite MBP epitope release in the damaged PNS of both sexes, the autoantibody circulated in the serum of female rats but not male rats. This data suggests that the sexual dimorphism lies downstream of MBP proteolysis and peptide release. In agreement, MBP-releasing MMPs (Liu et al., 2012; Ko et al., 2016; Hong et al., 2017; Chernov et al., 2018) exhibited comparable activity in female and male CCI nerves (Remacle et al., 2018b) and female-specific allodynia developed after an equal-dose MBP peptide injection (Chernov et al., 2020). The MBP peptide caused transcriptional reprogramming of the injected nerve and corresponding DRG and spinal cord through sex-specific changes in immune, metabolic, and nociceptive signaling through co-activation with estrogen receptor-1 (ESR1) (Chernov et al., 2020). In contrast to females, T-/B-cell-related gene activity was localized to the nerve, with no evidence for DRG activity (Chernov et al., 2020). It is important to emphasize that ESR activation by dietary intake of the synthetic estradiol stimulates T cells and IgM-positive B-cell expansion in females (Rodenas et al., 2017). Ultimately, the pro-allodynic MBP(84–104) activity is T-cell-dependent, as shown using athymic nude rats lacking mature T cells and by mutagenesis of the T-cell-binding site of the peptide (Liu et al., 2012; Chernov et al., 2018). The pro-allodynic effect of the rat-specific peptide sequence in rats (Hong et al., 2017) further supports an auto-reactive mechanism of MBP action in pain.

Many pain syndromes, such as CRPS, fibromyalgia (Fillingim et al., 2009; Nahin, 2015), and painful autoimmune diseases (Whitacre et al., 1999; Voskuhl, 2011), affect women with a significantly higher incidence than men. This preclinical study supports our finding of anti-MBP autoantibody in a subgroup of women with fibromyalgia and women with painful multiple sclerosis (Remacle et al., 2018a). Our present and earlier finding (Liu et al., 2012; Chernov et al., 2018; Remacle et al., 2018a) of the released MBP epitopes in morphologically intact myelin structures suggests their potential contribution to idiopathic pain states. IgM and IgG against MBP(84–104) were detected in the serum of women (Remacle et al., 2018a). Rat serum post-CCI was only reactive for IgM, the first antibody in response to the specific antigen (Sathe and Cusick, 2021), although evidence for IgG was detectable after PNS injury in mice (unspecified sex) (Vargas et al., 2010). The function of the autoantibody in persistent pain may be mediated by the respective IgG-Fc/IgM-Fcμ receptor activation on primary afferents (Xiao et al., 1997; Sorkin et al., 2002; Bennett and Vincent, 2012; Klein et al., 2012; McMahon et al., 2015; Goebel, 2016; Mifflin and Kerr, 2016; Wigerblad et al., 2016). IgM deficiency mitigates mechanical allodynia associated with a murine tibial fracture and cast immobilization model of CRPS, as IgM replacement from wild-type fracture mice resumed the pain-like behavior (Li et al., 2014, 2018). Based on the sequence homology between MBP(84–104) and M2 acetylcholine receptor, anti-MBP autoantibodies may contribute to CRPS (Shubayev et al., 2018).

Several lines of evidence support an autoantibody-independent MBP action in pain. The rapid onset allodynia after MBP(84–104) injection (hours to days) (Liu et al., 2012; Ko et al., 2016; Hong et al., 2017; Chernov et al., 2018, 2020) corresponds to no detectable IgM/IgG (data not shown) and contrasts the extended process of IgM/IgG generation (normally, several weeks), including that of anti-MBP(84–104) IgM post-CCI. Unilateral allodynia in the presence of systemic IgM post-CCI suggests an autoantibody-independent or a localized MBP action. This model corroborates our hypothesis of localized MBP action in damaged myelinated (inherently, mechanosensitive A-type) afferents based on myelin-specific interactors, thus sparing unmyelinated (inherently, nociceptive C-type) afferents, correlating with mechanical, not heat, hypersensitivity after MBP(84–104) injection (Liu et al., 2012; Ko et al., 2016; Shubayev et al., 2016; Chernov et al., 2020). The effect of a bolus IV RTX therapy on B-cell function without their depletion may relate to Th-cell activation and cellular calcium flux (Kazkaz and Isenberg, 2004), myelin debris clearance, and axon regeneration (Vargas et al., 2010). Lack of analgesic effect of IVIG immunotherapy in CCI-induced pain and pain in patients with CRPS (Goebel et al., 2010) favors this conclusion. In patients with IgM anti-MAG neuropathy, RTX mitigated pain and paresthesia through T reg cell activation (Dalakas et al., 2009), and in patients with multiple sclerosis, B-cell depletion caused inactivation of Th-cell activity in an autoantibody-independent manner (Stuve et al., 2005).

The effect of a bolus IV RTX (40 mg/kg) therapy in CCI-induced mechanical allodynia was female-specific albeit mild. This finding supports the mounting evidence for sex-specific adaptive immune arm activity in PNS trauma and mechanical allodynia in female vs. male rodents (Gregus et al., 2021). IV RTX, a mouse-human chimeric antibody, has shown to be effective in other diabetic rat models (58 mg/kg once weekly for 4 weeks) (Li et al., 2013) and adriamycin-induced (10 mg/kg once weekly) nephropathy (Takahashi et al., 2017) and hypertension (by infusion at 250 mg/kg) (Novotny et al., 2012). In addition to the CD20 monomer bands in CCI nerves of both sexes, an unexpected CD20-reactive 46 kDa band was selectively enriched in male nerves. Future studies using CD20 null animals and/or CD20 neutralization should help determine its specificity and origin (e.g., a post-translationally modified CD20). Regardless of the band’s identity, its male-specific reactivity with anti-CD20 detection antibody raises a possibility of its male-specific interference with RTX/anti-CD20 antibody therapy. B cell and splenocyte adoptive transfer, which potentiate mechanical allodynia in males post-CCI (Grace et al., 2011), could further study the role of sex and ESR activation in B-cell regulation of neuropathic pain.

Together, our findings support a model of a localized MBP-mediated autoimmune remodeling of sensory neuraxis in a subgroup of neuropathic pain cases associated with traumatic nerve injury. A serum anti-MBP autoantibody may serve as a biomarker of such painful states, particularly in women. Certain neuropathic pain states may be amenable to immunotherapeutic intervention in a selected cohort of female patients.

## Data Availability Statement

The original contributions presented in the study are included in the article/Supplementary Material, further inquiries can be directed to the corresponding author.

## Ethics Statement

The animal study was reviewed and approved by the Institutional Animal Care and Use Committee at the Veterans Affairs San Diego Healthcare System.

## Author Contributions

HL and JD conducted animal procedures. HL, JD, and SH performed the behavioral analysis. HL, JD, and JL carried out immunohistochemistry and immunoblotting analyses. AR developed and performed ELISA. VS conceived, designed, and directed the study. AS, AC, and TY contributed to study design, execution, and data analyses. HL and VS co-wrote the manuscript. All the authors read, edited, and approved the manuscript.

## Conflict of Interest

The authors declare that the research was conducted in the absence of any commercial or financial relationships that could be construed as a potential conflict of interest.

## Publisher’s Note

All claims expressed in this article are solely those of the authors and do not necessarily represent those of their affiliated organizations, or those of the publisher, the editors and the reviewers. Any product that may be evaluated in this article, or claim that may be made by its manufacturer, is not guaranteed or endorsed by the publisher.

## Supplementary Material

Supplementary Figure 1 Nerve and spleen CD20 levels were not changed after a bolus IV RTX. **(A)** CD20 immunoblotting (33, 46 kDa) in sciatic nerve and spleen (50 μg of protein) after IV-PBS (10 μl) or IV-RTX (10 mg/kg in 10 μl PBS) at day 17 post-CCI, upon completion of behavioral testing in Figure 2. β-actin, loading control. Representative of *n* = 3/group.

## Abbreviations

AUC: area under the curve
CCI: chronic constriction injury
CRPS: complex regional pain syndrome
DAPI: 4′,6-diamidino-2-phenylindole
ELISA: enzyme-linked immunosorbent assay
IV: intravenous
IVIG: intravenous immunoglobulin
MAG: myelin-associated glycoprotein
MBP: myelin basic protein
PNS: peripheral nervous system
RTX: Rituximab
SCR: scrambled
TMB: a 3,3′,5,5′-tetramethylbenzidine substrate.
