# Supplementary figures and images for: Sex-Specific B Cell and Anti-Myelin Autoantibody Response After Peripheral Nerve Injury
Source: Front Cell Neurosci. 2022 Apr 14;16:835800. doi: 10.3389/fncel.2022.835800 (PMC9050049; doi:10.3389/fncel.2022.835800)

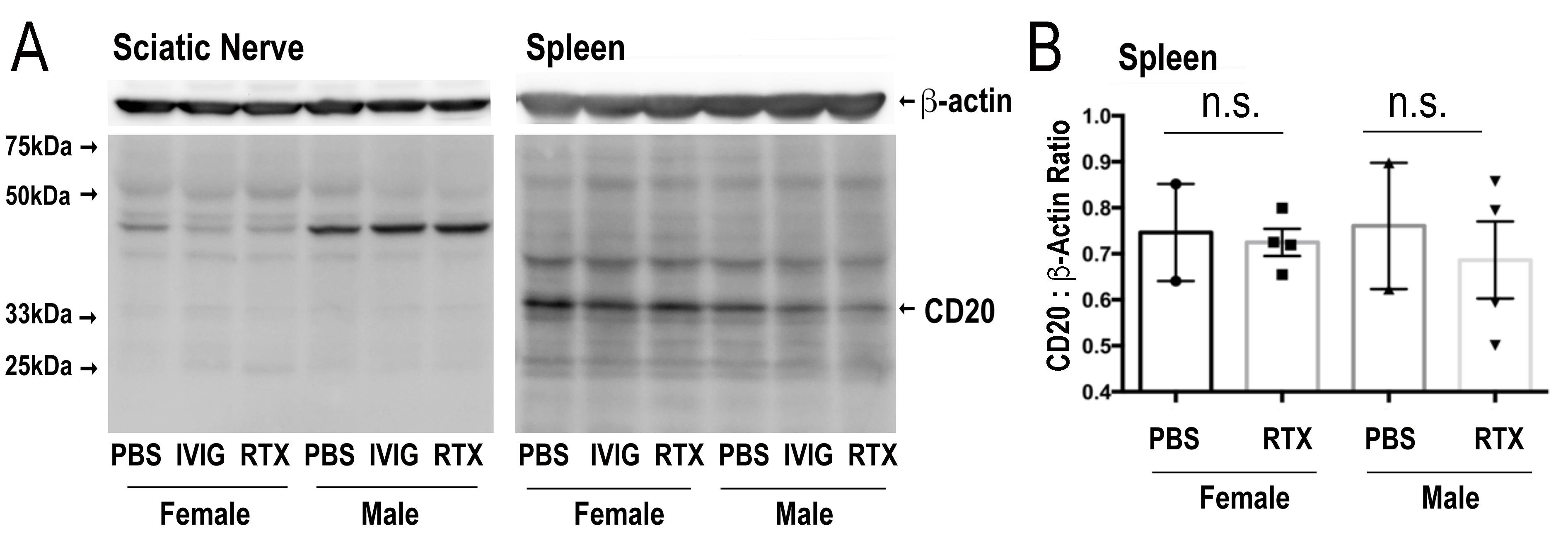

Supplement: Supplementary Figure 1 — Nerve and spleen CD20 levels were not changed after a bolus IV RTX. (A) CD20 immunoblotting (33, 46 kDa) in sciatic nerve and spleen (50 μg of protein) after IV-PBS (10 μl) or IV-RTX (10 mg/kg in 10 μl PBS) at day 17 post-CCI, upon completion of behavioral testing in Figure 2. β-actin, loading control. Representative of n = 3/group. [file Image_1.tif]
